# Phenotypic and genetic diversity in *Sinorhizobium meliloti *and *S. medicae *from drought and salt affected regions of Morocco

**DOI:** 10.1186/1471-2180-10-15

**Published:** 2010-01-20

**Authors:** Nadia Elboutahiri, Imane Thami-Alami, Sripada M Udupa

**Affiliations:** 1 Institut National de la Recherche Agronomique (INRA), Centre Régional de la Recherche Agronomique de Rabat, B.P. 415, Rabat, Morocco; 2 ICARDA-INRA Cooperative Research Project, International Center for Agricultural Research in the Dry Areas (ICARDA), B.P. 6299, Rabat, Morocco

## Abstract

**Background:**

*Sinorhizobium meliloti *and *S. medicae *are symbiotic nitrogen fixing bacteria in root nodules of forage legume alfalfa (*Medicago sativa *L.). In Morocco, alfalfa is usually grown in marginal soils of arid and semi-arid regions frequently affected by drought, extremes of temperature and soil pH, soil salinity and heavy metals, which affect biological nitrogen fixing ability of rhizobia and productivity of the host. This study examines phenotypic diversity for tolerance to the above stresses and genotypic diversity at Repetitive Extragenic Pallindromic DNA regions of *Sinorhizobium *nodulating alfalfa, sampled from marginal soils of arid and semi-arid regions of Morocco.

**Results:**

*Rsa*I digestion of PCR amplified 16S rDNA of the 157 sampled isolates, assigned 136 isolates as *S. meliloti *and the rest as *S. medicae*. Further phenotyping of these alfalfa rhizobia for tolerance to the environmental stresses revealed a large degree of variation: 55.41%, 82.16%, 57.96% and 3.18% of the total isolates were tolerant to NaCl (>513 mM), water stress (-1.5 MPa), high temperature (40°C) and low pH (3.5), respectively. Sixty-seven isolates of *S. meliloti *and thirteen isolates of *S. medicae* that were tolerant to salinity were also tolerant to water stress. Most of the isolates of the two species showed tolerance to heavy metals (Cd, Mn and Zn) and antibiotics (chloramphenicol, spectinomycin, streptomycin and tetracycline). The phenotypic clusters observed by the cluster analysis clearly showed adaptations of the *S. meliloti *and *S. medicae *strains to the multiple stresses. Genotyping with rep-PCR revealed higher genetic diversity within these phenotypic clusters and classified all the 157 isolates into 148 genotypes. No relationship between genotypic profiles and the phenotypes was observed. The Analysis of Molecular Variance revealed that largest proportion of significant (P < 0.01) genetic variation was distributed within regions (89%) than among regions (11%).

**Conclusion:**

High degree of phenotypic and genotypic diversity is present in *S. meliloti *and *S. medicae *populations from marginal soils affected by salt and drought, in arid and semi-arid regions of Morocco. Some of the tolerant strains have a potential for exploitation in salt and drought affected areas for biological nitrogen fixation in alfalfa.

## Background

The gram-negative bacteria *Sinorhizobium meliloti *and *S. medicae *are able to interact with roots of *Medicago sativa *(alfalfa) to form nitrogen-fixing nodules and survive as a free living saprophytic bacterium in the soil [1,2]. The host, alfalfa is the most important forage legume crop in the arid and semi-arid areas of North Africa. In these areas, alfalfa is grown in marginal soils and frequently subjected to abiotic and biotic stresses can affect both alfalfa and its nitrogen-fixing symbiotic bacteria in the root nodules [3].

In recent years, due to the reduced need for application of nitrogenous fertilizers, the rhizobia have gained a great agricultural value and play an important role in improving soil fertility in farming systems [3]. Inoculation of alfalfa with efficient strains of the rhizobia has significant economical and ecological benefits [3]. However, the presence of natural strains of rhizobia in the soils, usually highly competitive and well adapted to certain environment can reduce the inoculation benefits even with highly efficient strains. In addition, especially in marginal soils of arid and semi-arid regions, survival and effective functioning of natural and inoculated rhizobia populations are reduced by high soil temperatures, salt and osmotic stress, soil acidity, alkalinity and heavy metals in soils [3]. Added to this challenge, the rhizobia must cope with above abiotic stresses and they must survive as saprophyte and persist in such marginal soils in the absence of host plants [1]. Thus, knowledge about the diversity in natural population pertaining to above stresses is necessary before the selection and application of the tolerant strains of rhizobia for biological nitrogen fixation.

Although, phenotypic and genotypic diversity of some species of rhizobia are available [2,4-6], little is known about such diversity in natural populations of *Sinorhizobium *nodulating alfalfa in the marginal soils of arid and semi-arid regions, which are affected by salinity and frequent droughts. Thus, it is important to investigate the phenotypic and genotypic diversity and genetic structure of natural populations of the rhizobia in the marginal soils.

The use of molecular techniques has facilitated the development of rapid and simple methods for genetic diversity and genetic structure analysis of natural microbial populations. Studies utilizing restriction fragment length polymorphism-PCR, multilocus enzyme electrophoresis, 16S ribosomal DNA analysis, repetitive extragenic palindromic-PCR (rep-PCR), and DNA re-association have revealed extensive genetic variability of microbial communities in soils [4,7-13]. The rep-PCR method is more versatile and efficient than other methods for fingerprinting of bacterial isolates [14]; the generated PCR fingerprints are unique to each isolate in *S. meliloti *and group them at the strain level [15]. The variation determined with REP- and ERIC-PCR [15] was also shown to correlate with the measurement of genetic diversity using data generated by multilocus enzyme electrophoresis [16]. Thus, REP- and ERIC-PCR methods are very useful for genetic diversity and population genetic structure analysis of *Sinorhizobium *nodulating alfalfa.

In this study, we have sampled *Sinorhizobium *isolates nodulating alfalfa from marginal soils affected by salt and frequent droughts in arid and semi-arid regions of Morocco where alfalfa is being grown. The objectives of our work were: firstly, to characterized phenotypic diversity of the sampled isolates for tolerance to water and salinity stresses, extremes of temperature and pH, heavy metals and antibiotics *in vitro; *secondly, to estimate genetic diversity and genetic structure of the rhizobia populations in marginal soils of arid and semi-arid regions of Morocco; and finally, to relate the phenotypic and genotypic diversity in order to study whether the isolates within a phenotypic cluster derived from a single or very few lineages.

## Results and Discussion

### High degree of phenotypic diversity in the rhizobia populations from marginal soils

In this study we found that alfalfa in Morocco is nodulated by *S. meliloti *and *S. medicae*. Out of 157 sampled isolates, 136 and 21 isolates were identified as *S. meliloti *and *S. medicae*, respectively. *S. medicae *isolates were observed only in the samples collected by soil trapping method.

**Figure 1:**
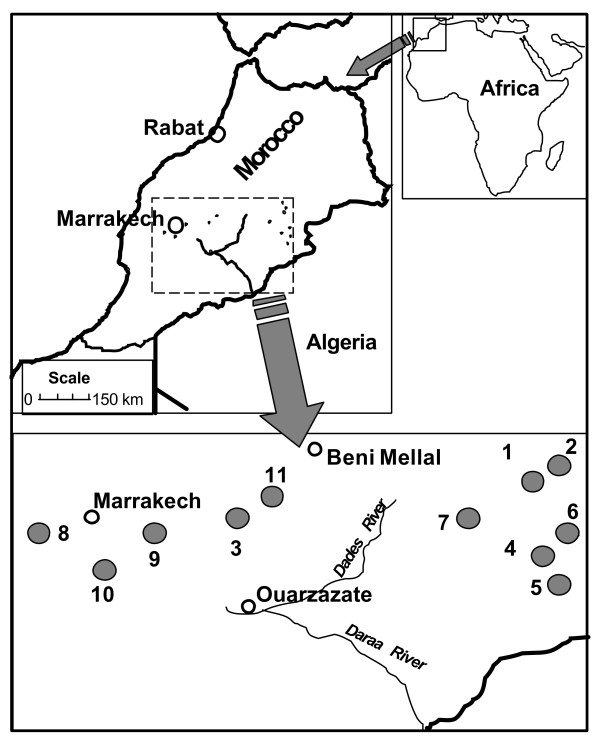
**A map showing sampling regions (closed circles)**. The numbers indicates different sampling regions: 1) Rich Errachidia, 2) Ziz, 3) Demnate, 4) Jerf Erfoud, 5) Rissani, 6) Aoufouss, 7) Tinghir, 8) Chichaoua, 9) Alhaouz, 10) Tahanaoute, and 11) Azilal.

Marginal soil is a complex environment where rhizobia growth and development can be influenced by several environmental stresses. Among them, salinity and water stresses, high temperature and pH and heavy metal stresses are very important; and are prevalent in alfalfa growing regions of Morocco (Figure 1; Table 1).

**Table 1:** Mean rainfall, temperature and soil properties in the sampling sites

Origin/population	Region	Isolate serial #	Mean rainfall (mm)^a^	Mean temperature^a^		Soil properties				
				
				Min. (°C)	Max. (°C)	pH range	EC range (ds/m)^b^	Mn (mg/Kg soil)^c^	Zn (mg/Kg soil)^d^	Cd (mg/Kg soil)^e^
Rich Kser Wallal	Rich Errachidia	1-11	260	-2.5	40	8.03-8.08	4.66-5.37	1.12	4.6	0.02
Rich Kser Aït Said	Rich Errachidia	12-20	260	-2.5	40	8.03-8.53	3.62-5.66	1.12	4.6	0.02
Rich Kser Tabia	Rich Errachidia	21-32	260	-2.5	40	8	5.51-7.18	1.12	4.6	0.02
Ziz Kser Tamgroutte	Ziz	33-39	130	0.5	42	8.04	6.36	0.98	3.2	0.02
Demnate	Demnate	40-56	480	0	35	7.77-8.10	6.26-7.40	1.58	5.2	0.02
Ziz Kser Bouya Jerf	Jerf Erfoud	57-58	75	1	45	nt	nt	nt	nt	nt
Jerf	Jerf Erfoud	59-67	75	1	45	8.09	5.39	0.86	3.2	0.06
Erfoud Kser Ouled Maat Allah	Jerf Erfoud	68-72	75	1	45	8.35	10.5	4.12	3.1	0.08
Erfoud Hay Lagmbita	Jerf Erfoud	73-88	75	1	45	7.97-8.43	3.97-5.20	4.12	3.1	0.08
Erfoud Masoudia	Jerf Erfoud	89-102	75	1	45	8.01	5.66	4.12	3.1	0.08
Rissani Kser Moulay Abdelleah	Rissani	103-104	60	0	50	nt	nt	nt	nt	nt
Rissani Mezguida	Rissani	105-107	60	0	50	nt	nt	nt	nt	nt
Errachidia Domaine Experimental	Rich Errachidia	108-109	120	-5	45	nt	nt	nt	nt	nt
Errachidia Aïne Zerka	Rich Erracidia	110-117	120	-5	45	8.24	6.06	1.64	5.1	0.08
Aoufouss Zaouit Amelkis	Aoufouss	118	120	-5	40	nt	nt	nt	nt	nt
Toudra Tinghir	Tinghir	119-121	250	-0.5	42	8.1	5.12	2.07	9.4	0.04
Ziz Errachidia	Ziz	122-129	130	0.5	42	nt	nt	nt	nt	nt
Ziz Erfoud	Ziz	130-136	130	0.5	42	nt	nt	nt	nt	nt
Rich Ziz	Ziz	137-145	130	0.5	42	nt	nt	nt	nt	nt
Chichaoua Mjjat	Chichaoua	146	240	4.9	39	7.33	4.5	2.52	6.2	0.08
Alhaouz Asni	Alhaouz	147-149	230	2	39	7.53	5.2	1.66	9.3	0.02
Tahanaout	Tahanaoute	150-152	250	4	42	7.51	3.52	1.9	5.1	0.02
Alhaouz Tahanaout Imgdal	Tahanaoute	153	250	4	42	7.23	6.09	1.9	5.1	0.02
Azilal Demnate Lahrouna	Azilal	154-157	130	-1	42	7.73-8.21	5.89-5.97	1.75	4.5	0.02

ppm = mg/Kg soil

^a^Average data of 15 year as of year 2005

nt = Not tested

Soil test interpretations (according to information available at http://ag.arizona.edu/crops/cotton/soilmgt/saline_sodic_soil.html; http://aces.nmsu.edu/pubs/_a/a-122.html; Personal communication by Dr Abdelmajid Zouahri, INRA, CRRA, Rabat, Morocco):

^b^For EC: Saline soil = EC > 4 ds/m; Normal soil = EC < 4 ds/m

^c ^For Mn: low = <1.0 mg/Kg; moderate = 1.0-2.5 mg/Kg; high = >2.5 mg/Kg

^d ^For Zn: low = <0.5 mg/Kg; moderate = 0.5-1.0 mg/Kg; high = >1.0 mg/Kg

^e ^For Cd: all the soils samples above the normal level (0.01 mg/Kg of soil)

**Figure 2:**
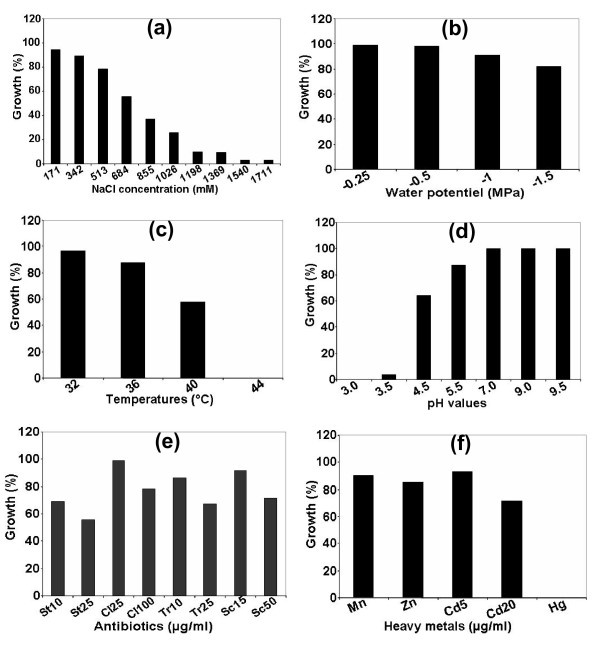
**Growth of isolates under salinity (a), water stress (b), high temperature (c), under different pH (d); and their resistance to antibiotics**. St: streptomycin; Cl: Chloramphenicol; Tr: Tetracycline; Sc: Spectinomycin and Concentrations: 10, 15, 25, 50 and 100 μg/ml (e), and heavy metals (Mn 300 μg/ml; Zn, 200 μg/ml; Hg, 20 μg/ml and Cd 5 and 20 μg/ml) (f).

The phenotypic characterization of the sampled 157 isolates for above characters revealed a large degree of variation (Figure 2; Additional file 1).

**Figure 3:**
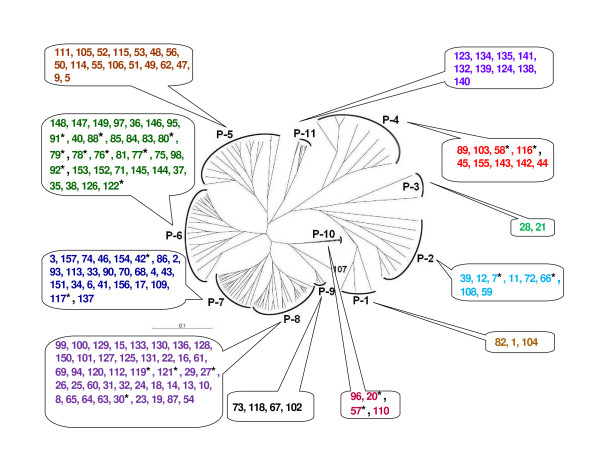
**Dendrogram showing relationships among *S. meliloti *and *S. medicae *isolates, based on phenotypic variation**. The UPGMA method was used for the cluster analysis. P-1 to P-11: phenotypic clusters. The numbers indicate *S. meliloti *isolate # and the numbers with asterisk (*) indicate *S. medicae *isolate #. Details of the individual clusters are presented in the text and Additional file 1.

Salinity is an important stress for rhizobia, because it inhibits persistence and development [17]. Consequently, a selection of rhizobia strains tolerant to salinity is of great importance for alfalfa cultivation in salt-affected areas. Indeed, after screening 157 isolates for salt tolerance, we observed a wide variability for tolerance at 171-1711 mM (1-10%) NaCl (Figure 2a); even isolates sampled from the same area/region showed variation for NaCl tolerance (compare Figure 3 and Table 2). 55.41% of the isolates (which includes 14 isolates of *S. medicae*) had good tolerance to NaCl (> 513 mM), indicating that the rhizobia nodulating alfalfa are more tolerant compared to other rhizobia species [3,18]. Four *S. meliloti *isolates (# 44, 45, 142 and 143) had greater tolerance to salt (1711 mM NaCl), which were sampled from the highly salt-affected areas of southern Morocco, than others, indicating that saline soils naturally select strains more tolerant to salinity, and results in higher recovery of salinity-tolerant strains.

**Table 2:** Sampling of *Sinorhizobium *isolates from drought and salt affected regions of Morocco

Origin/population	Region	Date of collection Month/Day/Year	Isolate serial #	Number of isolates collected		
				
				From nodules	From soil trapping	Total
Rich Kser Wallal	Rich Errachidia	8/4/2004	1-11	3	8	11
Rich Kser Aït Said	Rich Errachidia	8/4/2004	12-20	3	6	9
Rich Kser Tabia	Rich Errachidia	8/4/2004	21-32	3	9	12
Ziz Kser Tamgroutte	Ziz	8/4/2004	33-39	4	3	7
Demnate	Demnate	3/16/2005	40-56	10	7	17
Ziz Kser Bouya Jerf	Jerf Erfoud	8/5/2004	57-58	2	0	2
Jerf	Jerf Erfoud	8/6/2004	59-67	3	6	9
Erfoud Kser Ouled Maat Allah	Jerf Erfoud	8/5/2004	68-72	1	4	5
Erfoud Hay Lagmbita	Jerf Erfoud	8/5/2004	73-88	2	14	16
Erfoud Masoudia	Jerf Erfoud	8/5/2004	89-102	3	11	14
Rissani Kser Moulay Abdelleah	Rissani	8/5/2004	103-104	2	0	2
Rissani Mezguida	Rissani	8/5/2004	105-107	3	0	3
Errachidia Domaine Experimental	Rich Errachidia	8/6/2004	108-109	2	0	2
Errachidia Aïne Zerka	Rich Erracidia	8/6/2004	110-117	3	5	8
Aoufouss Zaouit Amelkis	Aoufouss	8/6/2004	118	1	0	1
Toudra Tinghir	Tinghir	8/6/2004	119-121	0	3	3
Ziz Errachidia	Ziz	4/30/1998	122-129	8	0	8
Ziz Erfoud	Ziz	5/8/1998	130-136	7	0	7
Rich Ziz	Ziz	6/17/1998	137-145	9	0	9
Chichaoua Mjjat	Chichaoua	3/3/2005	146	0	1	1
Alhaouz Asni	Alhaouz	3/10/2005	147-149	0	3	3
Tahanaout	Tahanaoute	3/10/2005	150-152	0	3	3
Alhaouz Tahanaout Imgdal	Tahanaoute	3/5/2005	153	0	1	1
Azilal Demnate Lahrouna	Azilal	3/16/2005	154-157	0	4	4

The isolates recovered displayed tolerance response to water stress, 82.16% of the isolates grew at water stress of -1.5 MPa (Figure 2b). Eighty isolates (which includes 13 isolates of *S. medicae*) that grew under salinity stress also grew under water stress. The common effect of salt and drought on rhizobia results in osmotic stress, which leads to changes in rhizobia morphology [19,20] and dehydration of cells. Some other authors [21,22] opined that the tolerant rhizobia accumulate osmolytes in response to the osmotic stress, which helps them to overcome effects of osmotic stress due to salinity and water stresses.

For the most rhizobia, optimum temperature range for growth of culture is 28-31°C, and many cannot grow even at 37°C [23]. At 28, 32 and 36°C, respectively, 100, 96.81 and 87.26% of the isolates grew well (Figure 2c). However, at 40°C, only 57.96% of the isolates (including 16 isolates of *S. medicae*) grew and these highly tolerant isolates were sampled from hot and dry regions of southern Morocco (Figure 1; Table 1; see Methods), complementing the similar observations made in cowpea rhizobia [24], in which they suggested that sampling from hot and dry areas facilitate selection for high temperature tolerance from the natural rhizobia populations.

There was a varied response of the isolates tested to pH (Figure 2d). All the isolates tested grew in alkaline pH (pH 9 and 9.5). At very low pH (pH 3.5), only 3.18% of isolates grew normally. Our study further confirmed that the alfalfa rhizobia are acid-sensitive [23,25,26] and most isolates only tolerated acidity of pH 5.5-6.0 [27,28].

The sampled isolates showed good tolerance to heavy metals such as Mn, Zn and Cd (Figure 2f). The highest number of isolates grew well in 5 μg/ml Cd (92.99%), followed by 300 μg/ml Mn (90.44%) and 200 μg/ml Zn (85.35%); and the growth of almost all isolates was inhibited by Hg (0.69%). 17 isolates of *S. medicae *were tolerant to the heavy metals (Mn, Zn and Cd). Our study showed that *S. meliloti *and *S. medicae *were more tolerant to the heavy metals than the other rhizobia species [29]. Since, the soils in the sampling sites were high in these heavy metals content, they might have exerted selection pressure on the rhizobia population [30], resulting in evolution of more tolerant strains.

The evaluation of intrinsic resistance to antibiotics showed that most tested isolates (> 85%) had high resistance to streptomycin, tetracycline, chloramphenicol and spectinomycin (Figure 2e). However, the degree of resistance to antibiotics was higher than in other species of rhizobia [5,31], indicating that *S. meliloti *and *S. medicae *had higher levels of tolerance to these antibiotics.

Isolates with different phenotypes were observed within a sampling location. The cluster analysis based on phenotypic data further revealed that these isolates represented phenotypically diverse populations. The 157 isolates formed 11 clusters (clusters P-1 to P-11; Figure 3; for detailed phenotypic characteristics of individual clusters, see Additional file 1).

Cluster P-1 consisted of three isolates; with different areas of origin. All isolates grew at 40°C, in the medium supplemented with 5% NaCl (855 mM), were resistant to water stress (-1.5 MPa), and sensitive to heavy metals, streptomycin and tetracycline.

Cluster P-2 consisted of 8 isolates from seven different areas. These isolates had a diversity of salt tolerance. All isolates grew in neutral-alkaline pH; and showed good growth at water stress of -1.5 MPa.

Cluster P-3 consisted of only two isolates from the Rich (Kser Tabia) area, and were very sensitive to salinity, but resistant to water stress.

Cluster P-4 consisted of nine isolates from seven different areas. All isolates grew at 40°C, were highly resistant to salinity (8-10%, i.e. 1368-1711 mM of NaCl) and to water stress (-1.5 MPa).

Cluster P-5 consisted of 17 isolates that were sensitive to salinity stress, had a wide range of diversity for water tolerance, and were resistant to heavy metals and antibiotics.

Cluster P-6 consisted of 32 isolates. All grew at 40°C, were resistant to heavy metals, and sensitive to streptomycin. They also grew at pH 4.5-9.5 and in medium supplemented with 1-4% NaCl. These isolates had a wide range of water stress tolerance.

Cluster P-7 consisted of 25 isolates. All grew in medium supplemented with 6% NaCl, at water stress level of -1.5 MPa and were resistant to heavy metals and antibiotics.

Cluster P-8 consisted of 43 isolates that were resistant to heavy metals and to antibiotics. They grew at 32-40°C, 3-4% NaCl, and had good tolerance to water stress.

Cluster P-9 consisted of four isolates, sensitive to Zn and resistant to antibiotics. They could grow at neutral-alkaline pH, were tolerant to water stress and to 5% NaCl.

Cluster P-10 consisted of four isolates. All grew at 40°C, tolerant to salinity, water stress and were sensitive to heavy metals and streptomycin.

Cluster P-11 consisted of nine isolates that grew in medium supplemented with 3% NaCl, and had a wide range of tolerance to temperature, water stress and heavy metals. All isolates were sensitive to tetracycline.

The phenotypic patterns observed in the cluster analysis clearly showed tolerance to the multiple environmental stresses which are common in marginal soils of arid and semi-arid regions. This kind of phenotypic diversity observed in the rhizobia populations could offer selective advantages in survival and adaptation to these harsh environments.

### Genotyping with rep-PCR resolved phenotypic diversity in *S. meliloti *and *S. medicae*

Rep-PCR analysis of consensus sequences REP and ERIC, capable of amplifying repetitive and conservative elements diffused/dispersed in DNA, revealed high intraspecific diversity among the 157 isolates and classified the isolates into 148 genotypes. Among the genotypes, only three genotypes were observed 2 times and one genotype was found 3 times and the remaining genotypes were detected only once. These identical genotypes were considered as clones and these clonal isolates were found only in *S. meliloti*. Since, each genotype characterized by unique combination of rep-PCR profiles, these genotypes can be considered as different strains.

**Figure 4:**
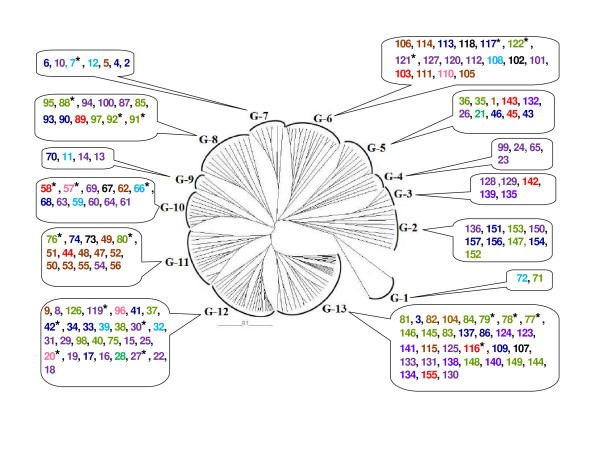
**Dendrogram showing genetic relationships among the isolates of *S. meliloti *and *S. medicae***. The UPGMA method was used for cluster analysis. G-1 to G-13: genotypic clusters. The isolates from the same phenotypic clusters (clusters P-1 to P-11, Figure 3) are denoted by the same colour, as shown in Figure 3. The numbers indicate *S. meliloti *isolate # and the numbers with asterisk (*) indicate *S. medicae *isolate #.

The dendrogram was constructed based on the genotype profiles and provided more information on the specific variability of the strains (Figure 4). At 84% level, there were 13 definitely separated and delimited clusters of strains. Each cluster contained strains with a range of phenotypic diversity. Each cluster was formed by strains from different areas of collection and with different phenotypic traits, except the cluster G-4 (all the 4 strains of the cluster with the same phenotype). In other words, within the same location/region of collection, the strains architecture was phenotypically and genetically divergent. Many strains belonging to various physiological groups (phenotypic clusters) were also distributed in various different (genotypic) clusters of the rep-PCR analysis, indicating they were genetically divergent isolates and there was no relationship between genetic profiles and phenotypes.

To study the extent of diversity at different rep-PCR loci, within sampling locations, regions and within phenotypic groupings, the genetic diversity index (GD) was estimated (Tables 3, 4, 5 and 6). The analysis showed that high genetic diversity for within sampling locations (GD ranged from 0.933 to 1.0) and within regions (GD ranged from 0.994 to 0.998; Table 5) for *S. meliloti*. For *S. medicae*, all the isolates were genetically different. Genetic diversity within phenotypic clusters for all the rhizobia were also high (GD ranged from 0.994 to 1.0; Table 6).

**Table 3:** Diversity estimates in *S. meliloti*

Origin	Region	Isolate serial number	Total number of isolates	Number of genotypes	Number of polymorphic loci	Polymorphic loci (%)	Genetic diversity
Rich Kser Wallal	Rich Errachidia	1-6; 8-11	10	10	28	75.68	1.00
Rich Kser Aït Said	Rich Errachidia	12-19	8	8	18	48.65	1.00
Rich Kser Tabia	Rich Errachidia	21-26; 28-29; 31-32	10	8	24	64.86	0. 933
Ziz Kser Tamgroutte	Ziz	33-39	7	7	14	37.84	1.00
Demnate	Demnate	40-41; 43-55	16	16	31	83.78	1.00
Jerf	Jerf Erfoud	59-65; 67	8	7	18	48.65	0.964
Erfoud Kser Ouled Maat Allah	Jerf Erfoud	68-72	5	5	27	72.97	1.00
Erfoud Hay Lagmbita	Jerf Erfoud	73-75; 81-87	10	10	27	72.97	1.00
Erfoud Masoudia	Jerf Erfoud	89-90; 93-102	12	12	31	83.78	1.00
Rissani Kser Moulay Abdelleah	Rissani	103-104	2	2	17	45.95	1.00-
Rissani Mezguida	Rissani	105-107	3	3	12	32.43	1.00
Errachidia Domaine Experimental	Rich Errachidia	108-109	2	2	10	27.03	1.00
Errachidia Aïne Zerka	Rich Errachidia	110-115	6	6	17	45.95	1.00
Aoufouss Zaouit Amelkis	Aoufouss	118	1	1	0	0	-
Toudra Tinghir	Tinghir	120	1	1	0	0	-
Ziz Errachidia	Ziz	123-129	7	6	20	54.05	0.952
Ziz Erfoud	Ziz	130-136	7	7	24	64.86	1.00
Rich Ziz	Ziz	137-145	9	9	23	62.16	1.00
Chichaoua Mjjat	Chichaoua	146	1	1-	-	-	-
Alhaouz Asni	Alhaouz	147-149	3	3	14	37.84	1.00
Tahanaout	Tahanaoute	150-152	3	3	21	56.76	1.00
Alhaouz Tahanaout Imgdal	Tahanaoute	153	1	1	0	0	-
Azilal Demnate Lahrouna	Azilal	154-157	4	4	21	56.76	1.00

**Table 4:** Diversity estimates in *S. medicae*

Origin	Region	Isolate serial number	Total number of isolates	Number of genotypes	Number of polymorphic loci	Polymorphic loci (%)
Rich Kser Wallal	Rich Errachidia	7	1	-	-	-
Rich Kser Aït Said	Rich Errachidia	20	1	-	-	-
Rich Kser Tabia	Rich Errachidia	27; 30	2	-	5	13.51
Demnate	Demnate	42	1	-	-	-
Ziz Kser Bouya Jerf	Jerf Erfoud	57-58	2	-	6	16.22
Jerf	Jerf Erfoud	66	1	-	-	-
Erfoud Hay Lagmbita	Jerf Erfoud	76-80; 88	6	6	20	54.05
Erfoud Masoudia	Jerf Erfoud	91-92	2	-	5	13.51
Errachidia Aïne Zerka	Rich Errachidia	116-117	2	-	9	24.32
Toudra Tinghir	Tinghir	119; 121	2	-	14	37.84
Ziz Errachidia	Ziz	122	1	-	-	-
Over all	-	-	21	21	35	94.59

**Table 5:** Analysis of population genetic structure using genotypic data of *S. meliloti*.

Regions/Groups	Number of populations	No. of genotypes	Genotypic diversity	Wright's F_ST _for haploids	Index of association (*I*_A_)	Sample size
Rich Errachidia	4	32	0.994**	0.267**	1.377**	34
Ziz	4	29	0.997**	0.203**	1.578**	30
Jerf Erfoud	4	34	0.998*	0.194**	0.854**	35
Over all (across populations)	12	95**	0.998**	0.250**	0.832**	99

*Significance at P < 0.05

**Significance at P < 0.01

**Table 6:** Genetic diversity within the phenotypic clusters of the rhizobia

Phenotypic cluster (P)	Number of isolates	Number of polymorphic loci	Number of genotypes	Genetic diversity
1	3	16	3	1.00
2	8	26	8	1.00
3	2	11	2	1.00
4	9	27	9	1.00
5	17	36	17	1.00
6	32	35	31	0.998
7	25	36	25	1.00
8	43	37	39	0.994
9	4	25	4	1.00
10	4	24	4	1.00
11	9	22	9	1.00

Exposure of alfalfa rhizobia to marginal soils with various stresses could have increased the phenotypic and genotypic diversity. It is possible that exposure of rhizobia to different niches of marginal soils which differ greatly in physical and chemical properties within soil complex may have resulted in evolution of wide diversity, which is necessary for their adaptation. The evolutionary processes [32] such as mutation, selection, gene flow/migration and recombination might have played a major role in the evolution of environmental stress tolerance and resulted in observed high diversity. Mutations generated variability; and marginal soil conditions and the host selected the adaptive variability in natural environments. Other processes like gene flow/migration and genetic exchange/recombination might have contributed to generation of a large number of genotypes with similar phenotypes.

Exposure of soybean rhizobia to stressful tropical environments had increased the number of rep-PCR profiles [33]; and exposure of clover rhizobia to toxic heavy metals resulted in evolution of diverse genotypes with many metal tolerance phenotypes [5], supported our findings.

It had been envisaged that tolerance to the environmental stresses such as salinity, osmotic stress, heavy metal toxicity and low pH is a complex process, involving many different genes present on chromosome and plasmids [5,34-36] and the stressful environment might have favored exchange, acquisition or modification of these genes, resulting in increased tolerance to the stresses.

We sampled both sensitive and tolerant types of rhizobia from marginal soils affected by salinity, drought, higher temperature and pH, and higher levels of heavy metals (Zn, Mn and Cd). Both sensitive and tolerant strains might have been maintained by the local cultivars of alfalfa by providing these rhizobia with protective niches (as reported for some other legumes [5,37]), thereby contributing to increased phenotypic and genotypic diversity. The rhizobia surviving in such microniches are further protected by their ability to invade roots and form symbiotic relationship with the plants.

### Spatial scale comparison of genetic structure

The differences in genetic structure of the rhizobia populations at regional levels were assessed by AMOVA. The largest proportion of significant (P < 0.01) genetic variation was found within regions (89%) than among the regions (11%), indicating regional subdivision of the genetic variability.

To study the extent of regional subdivision of the variability, population differentiation (measured by Wright's *F*_*ST*_) in some of the salinity and drought affected alfalfa growing regions of Morocco, was estimated only for *S. meliloti *populations with more than 5 isolates (i.e. for Rich Errachidia, Ziz and Jerf Erfoud regions only; Table 5). The population differentiation (Table 5) was moderate and ranged from 0.194 (P < 0.01; for Jerf Erfoud) to 0.267 (P < 0.01; for Rich Errachidia).

Very low percentage of clonal lineages and occurrence of a high degree of genetic variability among isolates observed in this study, suggesting that genetic recombination might have played an important role in generating new genotypes, which had profound influence on the genetic structure of natural populations.

Genetic recombination processes such as conjugation, transduction, and transformation allow the transfer of genes among rhizobia and may result in linkage equilibrium for their genes. However, many bacteria including some rhizobia species showed strong linkage disequilibrium [38-40]. To study linkage disequilibrium in *S. meliloti *populations, the index of association (*I*_*A*_) [39,41] was estimated (Table 5) for each region which consisted of 16 or more genotypes. A significant (P < 0.01) multilocus linkage disequilibria (LD) was observed for isolates from Rich Errachidia, Ziz and Jerf Erfoud regions, which apparently indicates restricted recombination between alleles at different loci. LD calculated (*I*_*A*_) for all the isolates was also significant. Strong linkage disequilibrium reflects either infrequent mixis of genotypes within local populations or results instead from limited migration between geographically isolated populations [42]. In our study, the regions which showed strong linkage disequilibrium also showed moderate population differentiation, suggesting that limited migration between populations and frequent mixis within populations in marginal environments contributed substantially to linkage disequilibrium in *S. meliloti *populations. In a previous study, exhibition of strong linkage disequilibrium in *Rhizobium leguminosarum *biovar *phaseoli *populations had been also attributed to limited migration between populations and frequent mixis within populations [42]. Selection for epistatic combinations of alleles at different loci can also maintain linkage disequilibrium in the face of frequent recombination, since such situations could be common under stressful environments, where rhizobia had to evolve for tolerance to multiple stresses.

## Conclusion

In this study, we observed that alfalfa in Morocco are nodulated not only by *S. meliloti *but also by *S. medicae*. We found high degree of phenotypic and genotypic diversity in *S. meliloti *and *S. medicae *populations from marginal soils affected by salt and drought, in arid and semi-arid regions of Morocco. Large molecular variability as reflected by rep-PCR analysis, was distributed within regions than between regions. It is possible that exposure of rhizobia to different niches of marginal soils which differ in physical and chemical properties within soil complex might have resulted in wide diversity we observed. The rhizobia isolates from the marginal soils of Morocco were genetically divergent and there was no relationship between genotypic profiles and the phenotypes. Some of the strains tolerant to salinity and water stresses have a potential for exploitation in salt and drought affected areas for biological nitrogen fixation in alfalfa. It has been shown that under drought stress, co-inoculation of leguminous plants with rhizobia and other plant-growth-promoting rhizobacteria resulted in augmented plant productivity and drought tolerance [43].

## Methods

### Isolate collection

The 157 rhizobia isolates used in this study were isolated either from nodules sampled in the field or from root nodules of young alfalfa plants grown in soil samples collected from the drought and salt affected areas of southern Morocco (isolated by a trapping method using the same local cultivar grown in the sampling sites; Tables 1 and 2; Figure 1). The collected soil samples were also analyzed for Electrical conductivity (EC), pH and metal content (Zn, Mn and Cd) using standard procedures http://ag.udel.edu/EXTENSION/agnr/soiltesting.htm; http://aces.nmsu.edu/pubs/_a/a-122.html. In these sampling locations, farmers grow local cultivars of alfalfa in olive orchards and depended on natural populations of rhizobia for nitrogen fixation. Rhizobia were isolated using standard procedures [44] from all the collected nodules. Single colonies were picked and checked for purity by repeated streaking and microscopic examination. All isolates were incubated at 28°C and maintained on Yeast Mannitol agar slants at 4°C, or in 20% (v/v) glycerol at -70°C. All 157 isolates were Gram-negative, fast-growing rhizobia, formed single colonies with diameters of 2-3 mm within 2-3 days on Yeast Extract Mannitol agar (YEM) plates, and showed a positive reaction to the bromothymol blue test [45,46].

### Isolate phenotyping

All physiological tests were carried out on YEM plates, except for water stress. Petri dishes containing defined medium were subdivided into squares, which were inoculated with 10 μl of bacterial culture grown for 48 h in YEM broth. The following treatments (with three replications) were applied: salt tolerance [47] at 0-10% NaCl at increments of 1%; temperature tolerance at 28, 32, 36, 40 and 44°C; water stress imposed using PEG 6000 [20] in YEM broth at a level of -0.25, -0.5, -1 and -1.5 MPa; pH tolerance [47] at pH 3.0, 3.5, 4.5, 5.5, 7.0, 9.0 and 9.5 (Homopipes buffer 25 mM used for pH range of 3-5, and for pH range 9-9.5 [pKa 7.5 at 25°C] and the MES buffer used for pH range 5-7 [pKa 6.1 at 25°C]); and intrinsic antibiotic [47] and heavy metal tolerance [47] were determined on solid YEM medium containing the following filter-sterilized antibiotics or heavy metals (all μg/ml): chloramphenicol (25 and 100), spectinomycin (15 and 50), streptomycin (10 and 25) and tetracycline (10 and 25); CdCl_2_.2H_2_O (5 and 20), MnCl_2 _(300), HgCl_2 _(20) and ZnCl_2 _(200). After 7 days of incubation at 28°C, the bacterial growth was compared to controls.

### Isolate genotyping

Bacterial DNA was extracted by a simple boiling method. Bacteria were grown in TY agar [48] petri dishes at 28°C for 2 days. Cells were suspended in 25 μl of sterile distilled water and followed by 25 μl of freshly prepared lysis-buffer containing 0.1 N NaOH and 0.5% SDS. The mixture was boiled in a water bath for 15 min. Then, 200 μl of TE (10 mM Tris-HCl and 0.1 mM EDTA) was added to the mixture, which was then centrifuged for 15 min at 12,000 g. The supernatant formed by the aqueous phase that contained clear and suspended DNA was transferred to new sterile tubes.

For the rhizobia species assignment, the 16S rDNA gene of the isolates was amplified using primers fD1 and rD1 with an annealing temperature of 58°C and restricted with *Rsa*I. Based on *Rsa*I restriction pattern, the isolates were assigned to either *S. meliloti *or *S. medicate *[2,49,50], by comparing their pattern with the restriction pattern of the reference strains *S. meliloti *(USDA, NRRL-45) and *S. medicae *(ABT5).

PCR targeting repetitive DNA sequences (rep-PCR) such as repetitive extragenic palindromic sequences (REP) [51] and enterobacterial repetitive intergenic consensus sequences (ERIC) [52] were performed according to de Bruijn [15] with minor modifications. Since BOX primer did not reveal any polymorphism in *S. meliloti *[53], it was not used in this study. The amplification was carried out in tubes containing 25 μl of final reaction volume. The reaction mixture contained 2.5 μl of DMSO (100%), 14.65 μl of sterile distilled water, 2.5 μl of PCR buffer (10×), 1.25 μl of dNTPs (2 mM), 0.55 μl of REP primers [51] (Rep1 5'-IIIICGICGICATCIGGC-3' and Rep2 5'-ICGICTTATCIGGCCTAC-3'; 0.3 μg each) or 0.44 μl of ERIC primers [51] (Eric1 5'ATGTAAGCTCCTGGGGATTCAC-3' and Eric2 5'AAGTAAGTGACTGGGGTGAGCG-3'; 0.3 μg each) and 0.4 μl (2U) of *Taq *polymerase. After the addition of 2 μl (50 ng) of DNA, the reaction mix was placed on a thermocycler (Mastercycler, Eppendorf, Germany) and subjected to PCR cycles: 95°C for 7 min, followed by 35 cycles of 94°C for 1 min, 53°C for 1 min and 65°C for 8 min, and followed by final elongation at 65°C for 8 min. PCR amplified fragments were electrophoresed in an agarose gel (1.5%) and visualized using ethidium bromide staining.

### Data analysis

Comparison of all physiological traits was performed on the basis of growth (1) or no growth (0) for each of the isolate. Comparison of amplified DNA profiles for each of the primers was performed on the basis of the presence (1) or absence (0) of REP and ERIC fragments. The binary data was used for estimation of shared allele distance and the shared allele distance was further used for cluster analysis based on the unweighted paired-group method using arithmetic averages (UPGMA) using the software program PowerMarker Version 3.25 [54].

The Analysis of Molecular Variance (AMOVA) [55] was performed using GenAlEx version 6.1 software [56]. For regions, Wright's *F*_*ST *_for haploids was calculated [57,58]. Wright's *F*_*ST *_for haploids (*θ*), can take values between 0 (no differentiation between locations) and 1.0 (complete differentiation between locations) [59].

The index of association (*I*_*A*_), a measure of multilocus linkage disequilibrium, Wright's *F*_*ST *_for haploids and genetic diversity were estimated using the software MultiLocus 1.3 [60].

## Authors' contributions

NE isolated the cultures, performed phenotyping and genotyping of the isolates, and also contributed in drafting the manuscript. ITA did sampling of the isolates, contributed to conception and the outline of the study, supervised phenotyping and drafting the manuscript. SMU contributed to conception and the outline of the study, supervision of genotyping, data analysis and drafting of the manuscript. All authors read and approved the final manuscript.

## Supplementary Material

Additional file 1 **Phenotypic** characteristics of the phenotypic clusters Click here for file
