# Supplementary material for: Phenotypic and genetic diversity in Sinorhizobium meliloti and S. medicae from drought and salt affected regions of Morocco
Source: BMC Microbiol. 2010 Jan 20;10:15. doi: 10.1186/1471-2180-10-15 (PMC2823721; doi:10.1186/1471-2180-10-15)
Supplement: Additional file 1 — Phenotypic characteristics of the phenotypic clusters [file 1471-2180-10-15-S1.PDF]

Additional file 1: Phenotypic characteristics of the phenotypic clusters

Phenotypic cluster number[a]	Isolates number[b]	Phenotypic characteristics					
		Salinity[c]	Water stress[d]	Temperature stress[e]	Tolerant pH range	Heavy metals[f]	Antibiotics[g]
1	82, 1, 104	Tolerant	Tolerant	Tolerant	5.5-9.5	Sensitive	Sensitive to Streptomycin & Tetracycline, Resistant to others
2	39, 12, 7*, 11, 72, 66*, 108, 59	Tolerant	Tolerant	Tolerant	7-9.5	Sensitive	Variable response
3	28, 21	Sensitive	Tolerant	Tolerant	7-9.5	Sensitive	Sensitive to Streptomycin & Tetracycline, Resistant to others
4	89, 103, 58*, 116*, 45, 155, 143, 142, 44	highly Tolerant	Tolerant	Tolerant	4.5-9.5	Resistant	Sensitive to Streptomycin, Resistant to others
5A	111, 105	Sensitive	Tolerant	Tolerant	4.5-9.5	Resistant	Resistant to all
5B	52, 115	Sensitive	Tolerant	Tolerant	4.5-9.5	Resistant	Resistant to all
5C	53, 48	Sensitive	Sensitive	Tolerant	4.5-9.5	Resistant	Resistant to all
5D	56, 50, 114, 55, 106	Very Sensitive	Sensitive	Tolerant	4.5-9.5	Resistant	Resistant to all
5E	51, 49, 62, 47, 9, 5	Sensitive	Sensitive	Tolerant	4.5-9.5	Resistant	Resistant to all
5	111, 105, 52, 115, 53, 48, 56, 50, 114, 55, 106, 51, 49, 62, 47, 9, 5	Sensitive	Wide range	Tolerant	4.5-9.5	Resistant	Resistant to all
6A	148, 147, 149	Tolerant	Tolerant	Tolerant	5.5-9.5	Resistant	Sensitive to streptomycin,

							Resistant to others
6B	97, 38	Tolerant	Tolerant	Tolerant	4.5-9.5	Resistance	Resistant to all
6C	36, 146, 95, 91*, 40, 88*, 85, 84, 83, 80*, 79*, 78*, 76*, 81	Sensitive	Tolerant	Tolerant	4.5-9.5	Resistant	Sensitive to streptomycin, Resistant to others
6D	77*, 75	Sensitive	Sensitive	Tolerant	4.5-9.5	Resistant	Sensitive to streptomycin, Resistant to others
6E	98, 92*, 153, 152	Tolerant	Sensitive	Tolerant	4.5-9.5	Resistant	Sensitive to streptomycin, Resistant to others
6F	71, 145, 144, 37, 35, 38, 126, 122*	Tolerant	Tolerant	Tolerant	3.5-9.5	Resistant	Sensitive to streptomycin, Resistant to others
6	148, 147, 149, 97, 36, 146, 95, 91, 40, 88, 85, 84, 83, 80, 79, 78, 76, 81, 77, 75, 98, 92, 153, 152, 71, 145, 144, 37, 35, 38, 126, 122	171-684 mM	Wide range	Tolerant	4.5-9.5	Resistant	Sensitive to Streptomycin, Resistant to others
7	3, 157, 74, 46, 154, 42*, 86, 2, 93, 113, 33, 90, 70, 68, 4, 43, 151, 34, 6, 41, 156, 17, 109, 117*, 137	Tolerant	Tolerant	Tolerant	4.5-9.5	Resistant	Resistant to all
8	99, 100, 129, 15, 133, 130, 136, 128, 150, 101, 127, 125, 131, 22, 16, 61, 69, 94, 120, 112, 119*, 121*, 29, 27*, 26, 25, 60, 31, 24, 18, 14, 13, 10, 8, 65, 64, 63, 30*, 23, 19, 87, 54, 32	Tolerant	Tolerant	Tolerant	4.5-9.5	Resistant	Resistant to all

9	73, 118, 67, 102	Tolerant	Tolerant	Tolerant	7-9.5	Sensitive to Zn	Resistant to all
10	96, 20*, 57*, 110	Tolerant	Tolerant	Tolerant	4.5-9.5	Sensitive	Sensitive to Streptomycin, Resistant to others
11A	123, 134	Tolerant	Sensitive	Tolerant	5.5-9.5	Sensitive to Zn	Sensitive to tetracycline, Resistant to others
11B	135, 141	Tolerant	Tolerant	Tolerant	4.5-9.5	Resistant	Sensitive to tetracycline, Resistant to others
11C	132, 139	Tolerant	Tolerant	132: Tolerant, 139: Sensitive	4.5-9.5	Sensitive to Zn	Sensitive to tetracycline, Resistant to others
11D	124, 138, 140	Tolerant	Tolerant	Sensitive	4.5-9.9	Resistant	Sensitive to tetracycline, Resistant to others
11	123, 134, 135, 141, 132, 139, 124, 138, 140	513 mM	Wide range	Wide range	4.5-9.5	Wide range	Sensitive to Tetracycline, Resistant to others
Unclustred	107	Tolerant	Tolerant	Tolerant	4.5-9.5	Sensitive	Resistant to all

[a]The numbers indicate phenotypic cluster numbers and capital alphabets indicate the subclusters within the main cluster.

[b]The numbers indicate *S. meliloti* isolates and the numbers with asterisk (*) indicate *S. medicae* isolates.

[c]Salinity: Tolerant = >513 mM NaCl, sensitive = <513 mM NaCl

[d]Water stress: Tolerant = > -1.0 MPa, sensitive = < -1.0 MPa

[e]Temperature stress: Tolerant = > 32 °C, sensitive = < 32 °C.

[f]Heavy metals (µg/ml): $MnCl_2$ (300), $ZnCl_2$ (200), $CdCl_2$ (5 and 20), $HgCl_2$ (20).

[g]Antibiotics (µg/ml): streptomycin (10 and 25), tetracycline (10 and 25), chloramphenicol (25 and 100), spectinomycin (15 and 50)
